# Infectious Complications Following CD30 Chimeric Antigen Receptor T-cell Therapy in Adults

**DOI:** 10.1093/ofid/ofaf541

**Published:** 2025-09-18

**Authors:** Felicia Cao, Yueling Xiu, Michael Mohnasky, Jonathan S Serody, Paul Armistead, Gianpietro Dotti, Melody Smith, Jonathan Huggins, Julia Messina, Bhanu Ramachandran, Jennifer Saullo, Joseph Stromberg, Manish K Saha, Megan Walsh, Barbara Savoldo, Natalie Grover, Heather I Henderson, Tessa M Andermann

**Affiliations:** Divisions of Hematology and Oncology, Department of Medicine, University of North Carolina at Chapel Hill, Chapel Hill, North Carolina, USA; Gillings School of Public Health, University of North Carolina at Chapel Hill, Chapel Hill, North Carolina, USA; School of Medicine, University of North Carolina at Chapel Hill, Chapel Hill, North Carolina, USA; Division of Hematology, Department of Medicine, University of North Carolina at Chapel Hill, Chapel Hill, North Carolina, USA; Lineberger Comprehensive Cancer Center, University of North Carolina at Chapel Hill, Chapel Hill, North Carolina, USA; Division of Hematology, Department of Medicine, University of North Carolina at Chapel Hill, Chapel Hill, North Carolina, USA; Lineberger Comprehensive Cancer Center, University of North Carolina at Chapel Hill, Chapel Hill, North Carolina, USA; Lineberger Comprehensive Cancer Center, University of North Carolina at Chapel Hill, Chapel Hill, North Carolina, USA; Department of Microbiology and Immunology, University of North Carolina at Chapel Hill, Chapel Hill, North Carolina, USA; Division of Blood & Marrow Transplantation and Cellular Therapy, Department of Medicine, Stanford University, Stanford, California, USA; Division of Infectious Diseases, Department of Medicine, Duke University, Durham, North Carolina, USA; Division of Infectious Diseases, Department of Medicine, Duke University, Durham, North Carolina, USA; Division of Infectious Diseases, Department of Medicine, University of North Carolina at Chapel Hill, Chapel Hill, North Carolina, USA; Division of Infectious Diseases, Department of Medicine, Duke University, Durham, North Carolina, USA; Division of Infectious Diseases, Department of Medicine, University of North Carolina at Chapel Hill, Chapel Hill, North Carolina, USA; Division of Nephrology, Department of Medicine, University of North Carolina at Chapel Hill, Chapel Hill, North Carolina, USA; School of Medicine, University of North Carolina at Chapel Hill, Chapel Hill, North Carolina, USA; Lineberger Comprehensive Cancer Center, University of North Carolina at Chapel Hill, Chapel Hill, North Carolina, USA; Division of Hematology-Oncology, Department of Pediatrics, University of North Carolina at Chapel Hill, Chapel Hill, North Carolina, USA; Division of Hematology, Department of Medicine, University of North Carolina at Chapel Hill, Chapel Hill, North Carolina, USA; Lineberger Comprehensive Cancer Center, University of North Carolina at Chapel Hill, Chapel Hill, North Carolina, USA; Division of Infectious Diseases, Department of Medicine, University of North Carolina at Chapel Hill, Chapel Hill, North Carolina, USA; Lineberger Comprehensive Cancer Center, University of North Carolina at Chapel Hill, Chapel Hill, North Carolina, USA; Division of Infectious Diseases, Department of Medicine, University of North Carolina at Chapel Hill, Chapel Hill, North Carolina, USA

**Keywords:** cancer, CAR T-cell therapy, hematological malignancies, immunocompromised host, infections

## Abstract

Infections are increasingly recognized as a complication of chimeric antigen receptor T-cell (CAR-T) therapy however the incidence of infections after non-CD19 targeted CAR-T is not yet known. We report, for the first time, infectious complications after CD30 CAR T-cell treatment for patients with Hodgkin lymphoma and peripheral T-cell lymphoma.

We retrospectively evaluated all 64 adult patients with relapsed/refractory CD30+ lymphomas who received anti-CD30 CAR T-cells at a single institution between 2016–2021. We assessed microbiologically confirmed infections within 1 year after cell infusion, censoring for relapse. We calculated infection density (total infections per 100 patient-days-at-risk), and cumulative incidence of infection divided into time periods postinfusion (days 0–28, 29–90, and 91–365). We compared infectious outcomes to a concurrent cohort of CD19 CAR-T recipients (n = 50) at the same institution.

Infection density in the first year after CD30 CAR T-cell infusion was 0.131 per 100 patient-days-at-risk, with 17 patients developing 19 total infections including 15 mild, 3 moderate, and 1 severe infection (1-year cumulative incidence of 32%; 95% confidence interval [CI], 19–47]). Infections were primarily viral (30%; 95% CI, 17–44) and most common early after infusion. Far fewer infections were bacterial in CD30 CAR-T recipients (4.9%; 95% CI, 1.3–13), in contrast to the CD19 cohort in which bacterial infections predominated and were more severe.

Microbiologically confirmed infections, primarily with respiratory viruses, were most common in the first 28 days after CD30 CAR-T infusion and most were mild. Our findings may have implications for antimicrobial prophylaxis guidelines after CD30 CAR-T therapy.

## Teaser

Most infections after CD30 CAR-T infusion were mild compared to infections in a concurrent cohort of CD19 CAR-T recipients at the same institution. Viral infections were more common in the first 28 days after CD30 CAR-T, but bacterial infections predominated early after CD19 CAR-T.

Chimeric antigen receptor T-cell (CAR-T) therapy has revolutionized the treatment of relapsed and refractory hematological malignancies. The first Food and Drug Administration–approved CAR T cells targeted CD19, a surface glycoprotein expressed on all B cells, for treatment of B-cell malignancies including leukemia and lymphoma [1]. CD19 CAR-T initially approved for treatment of relapsed/refractory B-cell acute lymphocytic leukemia, are now the standard of care for patients with aggressive non-Hodgkin lymphoma who have progressed after first-line therapy [2–6].

Commonly recognized side effects of CAR-T therapy include cytokine release syndrome (CRS) and immune effector cell-associated neurotoxicity (ICANS) [7, 8]. However, infections are increasingly recognized as common and often severe complications following CAR-T therapy. Infections are the most frequent cause of nonrelapse-related mortality after CD19 CAR-T for patients with non-Hodgkin lymphoma [9, 10]. The incidence of clinically defined infections after CD19 CAR-T ranged from 30% to 70% within 1 year postinfusion, with 15%–30% of infections classified as severe or life-threatening [9–13]. Infections were most commonly seen in the first month after CD19 CAR-T therapy, with the highest incidence of bacterial infections during this period. Factors associated with infectious risk following CD19 CAR-T included incidence of CRS/ICANS and corticosteroid use [9–11].

In contrast, CD30 is transiently expressed by subsets of activated T and B cells and is highly expressed in specific hematological malignancies such as Hodgkin lymphoma and anaplastic large cell lymphoma [14–16]. In a multicenter study, our group characterized the outcome of patients receiving CD30 CAR-T therapy and found an overall response rate of 72% for patients with relapsed/refractory Hodgkin lymphoma at 6 weeks posttreatment, with 36% progression-free survival over 1 year [17]. Another ongoing clinical trial is evaluating efficacy of CD30 CAR-T for patients with relapsed/refractory peripheral T-cell lymphoma (NCT04083495).

The patient population receiving CD30 CAR-T differs substantially from those receiving CD19 CAR-T, as Hodgkin lymphoma tends to affect younger patients who receive less cytotoxic chemotherapy and more antibody–drug conjugate or immunotherapy-based treatments [18, 19]. Infectious risk after CD30 CAR-T therapy has not previously been characterized, including types of infections and their severity. Currently, antimicrobial prophylaxis for any CAR-T treatment is based on existing guidelines using data from patients receiving CD19 CAR-T therapy [20], and few data exist to inform prophylaxis after CAR T cells targeting different antigens [21–23]. Here, we sought to characterize infectious risk during the first year after CD30 CAR-T infusion, benchmarked against infectious outcomes following CD19 CAR-T infusion.

## METHODS

### Patient Cohort

Patients in this study were adults ≥18 years of age at the University of North Carolina at Chapel Hill (UNCCH) with relapsed or refractory CD30+ lymphomas as part of clinical trials NCT02690545, NCT03602157, NCT04083495, and NCT02663297. Of note, NCT02663297 investigated CD30 CAR T-cell treatment as consolidation after autologous hematopoietic stem cell transplant (HSCT) for CD30+ lymphoma (n = 16) [24]. In a sensitivity analysis, we excluded HSCT/CD30 patients to address the potential for increased infection risk in this subgroup. All patients who received CD30 CAR-T infusion (1 July 2016 through 31 July 2021) were included in this retrospective review. For comparison, we included all adult patients ≥18 years of age at UNCCH who received CD19 CAR-T infusion (1 April 2018 through 30 November 2021) either as a commercial product or as part of a clinical trial (NCT03016377, NCT03696784, or NCT03594162). Our aim was to include all patients receiving CAR-T products (CD30 or CD19) through 2021 despite CD30 CAR-T starting 2 years earlier at our institution, to ensure an adequate number of patients in our analysis. Consent was not required for inclusion in this study but was required for treatment with CD30 CAR-T therapy and for trial CD19 CAR T-cell products. Approval was obtained from the UNCCH institutional review board (IRB#20-2745, PI: Andermann).

### Treatment and Prophylaxis

Manufacture of CD30 CAR T cells was performed as described previously [17]. Patients received 1–200 × 10^6^ CD30-targeted CAR T cells for relapsed or refractory disease following lymphodepleting chemotherapy. Patients enrolled in NCT03602157 (12 patients) received CD30 CAR T cells and additional T cells coexpressing CD30 CAR and C-C Motif Chemokine Receptor 4. Most patients received antimicrobial prophylaxis consisting of valacyclovir, and trimethoprim/sulfamethoxazole starting at lymphodepletion (valacyclovir) or at day + 30 (trimethoprim-sulfamethoxazole) until 6 months to 1 year postinfusion or CD4 ≥ 200. Patients also received neutropenia prophylaxis with levofloxacin and fluconazole when absolute neutrophil count (ANC) was ≤0.5 × 10^9^ cells/L. The severity of CRS and ICANS were graded as described previously [25]. Patients with grade ≥2 CRS or ICANS were treated with tocilizumab (8 mg/kg per dose intravenously) and/or corticosteroids as per institutional standards and previously published guidelines [25].

### Data Collection and Infection Definitions

Patient information was manually extracted from the electronic medical record and entered into a REDCap database by at least 2 independent reviewers (M.M., F.C., J.S., M.S., B.R.). Discrepancies between reviewers were resolved through adjudication by the primary study investigator (T.M.A.). Information regarding infections and antibiotics was abstracted starting 30 days prior through 365 days after CAR T-cell infusion. Censoring for infections within 1 year after infusion occurred with relapse, next line of treatment, or at last contact with UNCCH, whichever was earliest.

Only microbiologically determined infections were included in this study, based on a positive culture or diagnostic test. One exception to this definition was the diagnosis of dermatomal varicella zoster virus (VZV), which was included without an available positive diagnostic test if presentation met criteria as determined by the treating clinician. Bloodstream and respiratory infections were adjudicated based on Centers for Disease Control and Prevention/National Healthcare Safety Network criteria [26]. Lower respiratory tract infection was determined by the presence of hypoxemia and infiltrates on chest imaging. Infection severity was defined as mild (grade 1), moderate (grade 2), or severe (grade 3) based on Blood & Marrow Transplant Clinical Trials Network 2023 criteria [20]. One-year all-cause mortality was evaluated based on reporting by the patients’ primary clinical team and was not censored for relapse. Infection-related causes of mortality include both clinically and microbiologically defined infections.

### Statistical Analysis

We primarily reported infection density which was calculated as total number of infection events per patient-days-at-risk multiplied by 100. Continuous variables are reported as median and range; categorical variables are reported as number and percentage. For comparing characteristics between patients with and without infections, the Kruskal-Wallis test was used for continuous variables and Fisher exact test was used for categorical variables. The univariate association between patient variables and 1-year infection risk among CD30 CAR-T patients was estimated using a log binomial model [27]. Continuous variables were compared above versus below the median for this analysis. In cumulative-incidence curves, patients contributed time from the date of infusion until the earliest of the following: first infection (overall and stratified by pathogen type), death, or administrative censoring at 365 days postinfusion. *P* values < .05 were considered statistically significant. Analyses were performed using R software version 4.3.2, with the *cmprsk* package used to estimate the cumulative incidence of infection.

## RESULTS

### Patient Characteristics

Sixty-four adult patients received CD30 CAR-T therapy at UNCCH from 2016 to 2021 (Table 1). Most patients (n = 50, 78%) had Hodgkin lymphoma and far fewer (n = 14, 22%) had peripheral T-cell lymphoma. The median age was 40.9 years (range, 18–77). This was a heavily pretreated patient population, with patients receiving a median of 4 lines of therapy (range, 2–17) before CAR-T infusion. Fifty-six patients (88%) received an HSCT before CAR T-cell treatment, with the vast majority being autologous transplants. Sixteen patients (25%) in this cohort were part of a clinical trial in which patients underwent an autologous HSCT followed by CD30 CAR-T infusion as consolidation (“HSCT/CD30” patients). On average, patients underwent HSCT a median of 1087 days before CD30 CAR-T (range, 16–6186 days). Infections within 30 days prior to CD30 CAR-T were minimal and occurred only in the group of patients who did not develop infections following CAR-T (Table 1).

**Table 1.** Comparison of Infected Versus Noninfected Patients Within 1 Year After Anti-CD30 CAR-T Therapy

Patient Characteristics	Infected	Noninfected	Total
N (%), Median [total range]	N = 17	N = 47	N = 64
Age	36.3 [18.0, 75.7]	41.3 [20.1, 77.0]	40.9 [18.0, 77.0]
Sex (male)	9 (52.9)	33 (70.2)	42 (65.6)
Race			
White	15 (88.2)	35 (74.5)	50 (78.1)
Black	2 (11.8)	5 (10.6)	7 (10.9)
Other	0 (0.0)	7 (14.9)	7 (10.9)
Ethnicity			
Hispanic	0 (0.0)	1 (2.1)	1 (1.6)
Non-Hispanic	17 (100.0)	42 (89.4)	59 (92.2)
Unknown	0 (0.0)	4 (8.5)	4 (6.3)
Malignancy			
Hodgkin lymphoma (HL)	13 (76.5)	37 (78.7)	50 (78.1)
Peripheral T-cell lymphoma	4 (23.5)	10 (21.3)	14 (21.9)
Disease state prior to CAR-T			
Complete remission (CR)	4 (23.5)	16 (34.0)	20 (31.3)
Partial remission (PR)	4 (23.5)	7 (14.9)	11 (17.2)
Stable disease (SD)	1 (5.9)	3 (6.4)	4 (6.3)
Progressive disease (PD)	8 (47.1)	21 (44.7)	29 (45.3)
KPS score	90.0 [80.0, 100]	90.0 [70.0, 100.0]	90.0 [80.0, 90.0]
HCT-CI score	2.0 [2.0, 5.0]	2 [2.0, 6.0]	2.0 [2.0, 6.0]
Prior lines of chemotherapy	5.0 [2.0, 10.0]	4.0 [2.0, 17.0]	4.0 [2.0, 17.0]
CAR-T lymphodepletion regimen			
Fludarabine/bendamustine	11 (64.7)	26 (55.3)	37 (57.8)
Fludarabine/cyclophosphamide	0 (0.0)	3 (6.4)	3 (4.7)
Bendamustine	3 (17.6)	4 (8.5)	7 (10.9)
Post-HSCT infusion	2 (11.8)	14 (29.8)	16 (25.0)
Other	1 (5.9)	0 (0.0)	1 (1.6)
HSCT before CAR-T	15 (88.2)	41 (87.2)	56 (87.5)
Allogenic	1 (5.9)	5 (10.6)	6 (9.4)
Autologous	14 (82.4)	36 (76.6)	50 (78.1)
Median duration between HSCT and CAR-T (D)	1111.0 [810.0, 1980.0]	969.0 [27.0, 1698.0]	1087.0 [16.0, 6186.0]
Auto-HSCT within 90 d of CAR T-cell infusion	2 (11.8)	14 (29.8)	16 (25.0)
HSCT within 1 y after CAR-T	0 (0)	0 (0)	0 (0)
Corticosteroids^a^	6 (35.3)	13 (27.7)	19 (29.7)
Before infection	2 (11.8)	–	2 (11.8)
Antimicrobial prophylaxis			
Fluoroquinolones	8 (47.1)	28 (59.6)	36 (56.3)
Fluconazole	9 (52.9)	28 (59.6)	37 (57.8)
Trimethoprim-sulfamethoxazole	6 (35.3)	23 (48.9)	29 (45.3)
Valacyclovir	12 (70.6)	36 (76.6)	48 (75.0)
ANC at day –30	2.5 [1.2, 5.2]	3.7 [0, 40.8]	3.4 [0.0, 40.8]
ALC at day –30	0.9 [0.2, 2.2]	0.7 [0.1, 3.4]	0.7 [0.1, 3.4]
ANC at lymphodepletion	2.8 [1.4, 5.8]	3.4 [1.0, 20.7]	3.2 [1.0, 20.7]
ALC at lymphodepletion	0.9 [0.1, 2.6]	0.6 [0.0, 3.3]	0.7 [0.0, 3.3]
Total days of neutropenia^b^	0.0 [0.0, 26.0]	0.0 [0.0, 33.0]	0.0 [0.0, 33.0]
Total days of lymphopenia^c^	7.0 [0.0, 15.0]	7.0 [0.0, 115.0]	7.0 [0.0, 115.0]
Infection within 30 d before CAR-T	0 (0.0)	3 (6.4)	3 (4.7)
Pre-CAR-T infection by organism			
Bacterial	0 (0.0)	2 (4.3)	2 (3.1)
Viral	0 (0.0)	1 (2.1)	1 (1.6)
Fungal	0 (0.0)	0 (0.0)	0 (0.0)
Time in study (d)	164 [22, 365]	183 [7365]	180 [7, 365]

Abbreviations: ALC, absolute lymphocyte count (×10^9^ cells/L); ANC, absolute neutrophil count (×10^9^ cells/L); HCT-CI, hematopoietic stem cell transplant-specific comorbidity index; HSCT, hematopoietic stem cell transplant; KPS, Karnofsky Performance Status.

^a^Steroids (30 days before CAR T through 1 year after CAR T or until relapse) include dexamethasone, prednisone, and methylprednisolone.

^b^Neutropenia defined as ANC <0.5 × 10^9^ cells/L.

^c^Lymphopenia defined as ALC <0.2 × 10^9^ cells/L.

The incidence of CRS and ICANS was similar to previously published data for CD30 CAR-T (Table 2) [17]. Sixteen patients (25%) who received CD30 CAR-T therapy experienced CRS of any grade, with only 5 patients (8%) demonstrating grade 2 CRS, and none with grade 3 or higher CRS. Only 5 patients (7.8%) received tocilizumab and none received steroids. No patients were diagnosed with ICANS. A total of 8 (12.5%) patients died within 1 year of infusion, primarily from relapse (62.5%). Three patients died from reported infection (37.5%), with 2 of these being suspected infections (ie, sepsis, hypoxemia) without a microbiological diagnosis (1 after relapse in the noninfected cohort, and 1 before relapse in the infected cohort). The third patient in the noninfected cohort died secondary to *Escherichia coli* bacteremia following disease relapse.

**Table 2.** CAR T-related Outcomes and Toxicity in Patients Within 1 Year After Anti-CD30 CAR-T Therapy

Patient Characteristics	CD30
N (%), Median [total range]	N = 64
Cytokine-release syndrome (CRS)	16 (25.0)
CRS grade	
1	11 (17.2)
2	5 (7.8)
3	0 (0)
CRS treatment	
Tocilizumab	5 (7.8)
Tocilizumab + steroids	0 (0)
Neurotoxicity (ICANS)	0 (0)
ICU admission (within 30 d of CAR-T infusion)	3 (4.7)
Relapse within 1 y after CAR-T cell therapy	37 (57.8)
30-day all-cause mortality	0 (0)
1-year all-cause mortality^a^	8 (12.5)
Cause of 1-year mortality^b^	N = 8
Infection-related	3 (37.5)
Relapse-related	5 (62.5)
Unknown	1 (12.5)

^a^Mortality regardless of relapse status.

^b^One patient had 2 causes of death listed (relapse and infection).

### Incidence of Infections After CD30 CAR T-cell Therapy

In our analysis, we included only microbiologically confirmed infections within 1 year following CD30 CAR-T infusion, censoring at the time of relapse. In the CD30 cohort, 17 patients (27%) developed 19 infections with 20 total organisms within 1 year after CAR-T infusion (Table 3). Of these infectious episodes, 3 were caused by bacteria (16%) and 16 by viruses (84%). Only 1 of these was a bloodstream infection. The majority of viral infections were respiratory viruses (n = 11, 57.9%). EBV viremia occurred in 2 patients with high viral titers thought to be unrelated to the underlying malignancy and requiring treatment (corticosteroids/rituximab). VZV rashes occurred in 3 patients (1 month, 2 months, and 3 months after CAR T infusion), none of whom were on antiviral prophylaxis at the time; mucocutaneous HSV-1 occurred in 1 patient on valacyclovir prophylaxis. No fungal infections were observed. The cumulative incidence of all infections within 1 year after CD30 CAR-T is shown in Supplementary Figure 1. Overall, the infection density in CD30 CAR-T recipients during this time period was 0.131 infections per 100 patient-days-at-risk. Excluding the 16 patients in the HSCT/CD30 group (3 viral infections), we observe an increase in the infection density to 0.162 per 100 patient-days-at-risk.

**Table 3.** Microbiologically Confirmed Infections Within 1 y After CD30 CAR T-cell Infusion

Type of Infection	Organism
Bloodstream infection	*Serratia marcescens*
Other disseminated infection	EBV (2)
Skin and soft tissue infection	
Cellulitis	*Staphylococcus aureus*
Shingles	VZV (3)
Mucocutaneous ulceration	HSV-1^a^
Pharyngitis	*Streptococcus dysgalactiae*
Respiratory tract infection	
Upper tract	Non-SARS coronavirus (2)^a^
	Rhinovirus (3)
	RSV (2)
	Influenza A^a^
	SARS-CoV-2 (2)
Lower tract	SARS-CoV-2

Abbreviations: EBV, Epstein-Barr virus; HSV, herpes simplex virus; RSV, respiratory syncytial virus; VZV, varicella zoster virus.

Type and number of bacterial and viral infections within 1 year after CD30 CAR-T infusion, censored for relapse. In total, 17 patients developed 19 episodes of infection with a total of 20 organisms. One patient had simultaneous RSV and Rhinovirus upper respiratory tract infections. Numbers in paratheses indicate total number of infections for causative organism. No fungal infections were observed.

^a^Occurred in patients who had received an auto-HSCT immediately before CD30 CAR-T.

**Figure 1.**
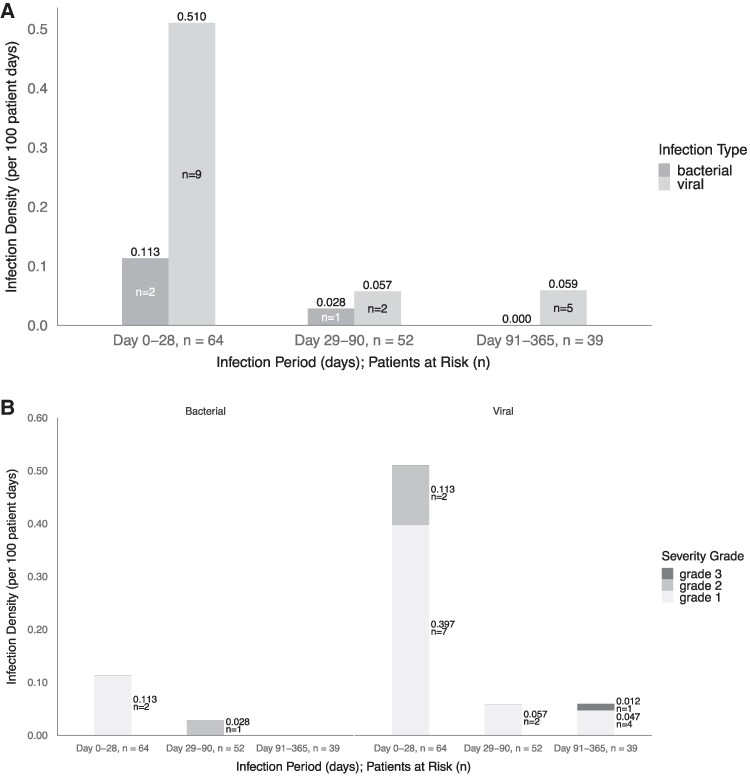
Increased density of mild viral infections observed within first 28 d after CD30 CAR-T. *A*, Density of bacterial and viral infections during the first 1 y following CAR T-cell therapy by infection periods (0–28, 29–90, and 91–365 d). Data shown are censored for relapse. No fungal infections were observed. *B*, Severity of infections including bacterial (left panel) and viral (right panel) occurring during the first 1 y following CAR-T therapy, divided into infection periods (0–28, 29–90, and 91–365 d) and censored for relapse.

The infection density of bacterial and viral infections from days 0–28, days 29–90, and days 91–365 for CD30 patients is shown in Figure 1*A*. Censoring occurred throughout the study due to relapse, leaving only 52 patients at days 29–90 and 39 patients at days 91–365 for analysis. Patients developed infections most commonly within the first 28 days following CAR-T infusion (median 28 days; interquartile range [IQR] 16.5–83.0). Viral infections were prevalent following CD30 CAR-T infusion at all timepoints, but most predominantly in the first 28 days following cell infusion. Three patients (4.7%) developed a respiratory tract infection with SARS-CoV-2, with a median of 265 days following CAR-T infusion (IQR 217.5–292) (Table 3). The severity of microbiologically confirmed infections within 1 year after CAR-T infusion was assessed using Blood & Marrow Transplant Clinical Trials Network 2023 criteria [20] (Supplementary Table 1). Most infections were caused by respiratory viral pathogens and were mild (Figure 1*B*; Supplementary Figure 2 with HSCT/CAR-T patients removed). Only 1 patient had a severe infection, in this case with SARS-CoV-2 lower respiratory tract infection (Supplementary Table 1).

### Factors Associated With Increased Infectious Risk

Baseline characteristics of the 17 patients who developed a microbiologically confirmed infection in the first year after CD30 CAR-T infusion were compared to the 47 patients who did not develop an infection (Table 1). In univariate analysis, only ANC at 30 days before cell infusion was significantly different between the infected and noninfected patients (relative risk, 0.31; 95% CI, 0.11–0.84; Supplementary Table 2). Antimicrobial prophylaxis use, including both antibacterials and antivirals, was similar between infected and noninfected patients (Table 1). Corticosteroid use before infection occurred in only 2 patients (11.8% of those infected), precluding consideration as a predictor of infection. The small number of patients in our study restricted further multivariable analysis.

### Immune Reconstitution After CD30 CAR T-cell Therapy

**Figure 2.**
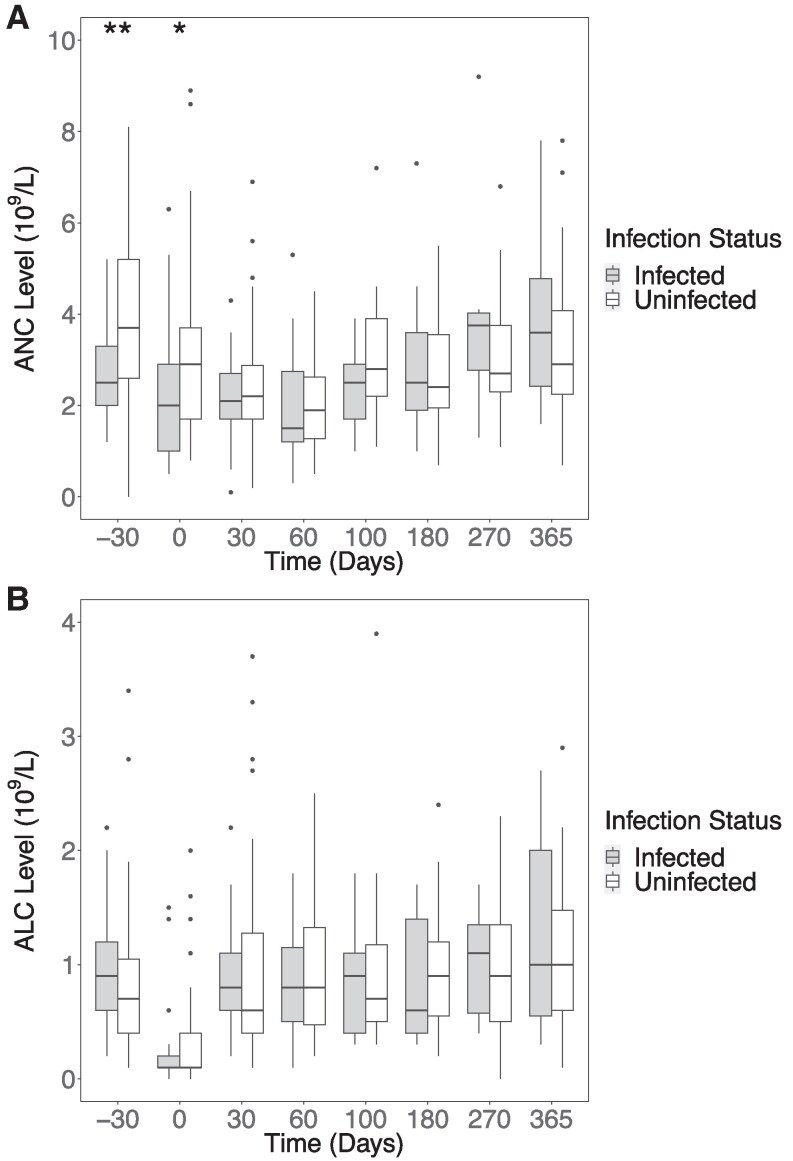
Immune recovery after CD30 CAR T-cell therapy demonstrates few differences between infected and uninfected patients within 1 y after infusion. *A*, Absolute neutrophil counts (ANC) and *B*, absolute lymphocyte counts (ALC) at timepoints relative to the day of CAR-T infusion. Comparisons between infected and uninfected patients were performed using the Wilcoxon rank-sum test. *P < .05, **P < .01. Note: Seven ANC outlier values are missing from the plot (range, 13.2–40.8) and 2 ALC outlier values are missing from the plot (range, 5–14).

Given the association between day 30 ANC and infectious complications in CD30 CAR-T patients, we sought to better understand the trajectory of immune reconstitution peri-infusion, both including (Figure 2) and excluding the HSCT/CAR-T cohort (Supplementary Figure 3). We examined the kinetics of absolute neutrophil and lymphocyte counts (ANC and absolute lymphocyte count [ALC]) 30 days before and at multiple timepoints after CAR T-cell infusion, highlighting differences in ANC and ALC between patients with and without infections (Figure 2). ANC levels 30 days before CD30 CAR T infusion and at day 0 were significantly lower in infected versus noninfected patients, with differences disappearing at subsequent timepoints through 1 year.

### Benchmarking Against CD19 CAR-T Recipients at the Same Institution

Although very different from the CD30 CAR-T patients, for the purposes of benchmarking, we included a cohort of all 50 patients treated with CD19 CAR-T at UNCCH from 2018 to 2021 (Supplementary Table 3). Benchmarking allowed us to: (1) determine whether viral infections were more predominant in CD30 patients due to increased respiratory pathogen testing during the SARS-CoV-2 pandemic or were more specific to CD30 CAR-T recipients relative to concurrent CD19 CAR-T recipients, and (2) provided a comparator group using the same methodology and definitions (prior studies are with CD19 CAR-T recipients and both microbiologically and clinically confirmed infections). Patients receiving CD19 CAR-T therapy were older, with a median age of 60.6 (range, 21–81) years as expected. They were similarly heavily pretreated relative to CD30 CAR-T patients, having also received a median of 4 (range, 2–12) prior treatment lines. The proportion of those in the CD19 CAR-T cohort who had received a prior HSCT (14 patients, 28%) was lower compared to CD30 CAR-T recipients (n = 56, 87.5%). Far more patients in the CD19 cohort received an allogeneic HSCT (57%) relative to CD30 CAR-T recipients (10.7%), although CD30 patients were more likely to have received an HSCT in the 90 days prior to CAR-T (45.3% v. 8%).

In contrast to our CD30 cohort, a higher number of patients (n = 34, 68%) in the CD19 group developed CRS of any grade, with 16 patients (32%) who developed grade 2 CRS and 1 patient (2%) who developed grade 3 CRS (Supplementary Table 4). Fourteen patients (28%) developed ICANS after CD19 CAR-T infusion. CRS treatment with tocilizumab +/− steroids was common after CD19 CAR-T infusion (n = 20, 40%). All-cause mortality was also higher after CD19 CAR-T infusion, occurring for 40% of patients (n = 20), and primarily from relapse (n = 15, 75%).

A smaller number of patients (n = 9, 18%) CD19 CAR-T patients developed microbiologically confirmed infections (infection density of 0.091 infections per 100 patient-days-at-risk) within 1 year from cell infusion (Supplementary Figure 4), censoring at the time of relapse. Median time to infection was 14 days (IQR 6–53). Of these infections, a higher proportion were bacterial (n = 6, 66.7%) than viral (n = 3, 33.3%) with 3 bacterial infections resulting from bacteremia and 2 from *Clostridioides difficile* colitis (Supplementary Table 5). No VZV, HSV-1, or fungal infections were observed. In comparison to the CD30 cohort, more infections in the CD19 group were caused by bacteria, most often in the first 28 days, and were higher grade, including 1 grade 3 bacterial infection (*Enterococcus faecalis* endocarditis; Supplementary Figure 4). In contrast to CD30 CAR-T recipients who infrequently received corticosteroids, 6 (66.7%) CD19 CAR-T patients received corticosteroids before infection, consistent with published data [9, 11, 13, 21, 22]. ANC and ALC in patients receiving CD19 CAR-T infusion are shown in Supplementary Figure 5, with few differences between infected and uninfected patients. Most notably, ANC levels decrease immediately after infusion and remain low for at least 30 days, compared to CD30 patients in whom significant neutropenia was not commonly observed.

## DISCUSSION

We present the first report of infectious complications in patients treated with CD30 CAR T-cell therapy for relapsed/refractory hematological malignancies. We found a higher incidence of viral infections compared with bacterial infections in patients treated with CD30 CAR-T therapy [9–11]. Given that many patients received CD30 CAR-T cell infusion during the SARS-CoV-2 pandemic, we compared CD30 outcomes with a cohort of all patients who had received a CD19 CAR T-cell product at our institution through the same year (2021). We found that bacterial infections were higher in CD19 CAR-T recipients, as previously reported [9–12], supporting our conclusion that higher viral infections after CD30 CAR-T did not likely reflect more frequent viral testing during the pandemic. Additionally, a greater proportion of infections occurred in the initial 28-day period after cell infusion for both groups. Unexpectedly, the higher early incidence of viral infections in the CD30 cohort did not appear to be related to lymphopenia: ALC both before CAR T and during lymphodepletion was notably low (median 0.7 × 10^9^ cells/L for both time points; Table 1) and no significant differences were observed between infected and noninfected groups (Figure 2). As lymphocyte subsets were not collected, it also remains unknown if particular lymphocyte subsets were differentially affected by CD30 CAR T cells. Only ANC 30 days before CAR-T infusion was significantly lower in infected patients in univariate analysis. This may be a spurious finding or a marker for a more heavily pretreated patient. Notably, ANC before lymphodepletion is included in the CAR HEMATOTOX score, which is predictive of severe infection after CD19 CAR-T therapy [28]. However, required elements of the CAR HEMATOTOX score (eg, C-reactive protein, ferritin) had not been collected in the majority of CD30 CAR-T patients, and we were unable to investigate its ability to predict infection in CD30 CAR-T recipients.

Prior work has found that both the incidence and treatment of CRS/ICANS are associated with increased infection risk after CD19 CAR-T therapy. As expected, the incidence of CAR-T–related toxicity was far less frequent and less severe in CD30 compared to CD19 patients (eg, no incidence of ICANS after CD30 CAR-T) and was not associated with infection in our study. Far fewer patients in the CD30 group received corticosteroids before infection compared to those in the CD19 group. However, most CD30 CAR-T patients had undergone prior HSCT (87.5%), including 16 patients who received autologous HSCT immediately before CD30 CAR-T infusion as part of a trial protocol. This additional immunosuppression in the CD30 group may explain why infections were more frequent, but not the reduced severity of infection compared to CD19 CAR-T recipients. Notably, removing patients in the HSCT/CAR T subgroup who were thought to be more immunosuppressed increased infection density. Most importantly, baseline disease risk rather than CAR T-cell therapy itself likely contributes more substantially to the differences in infection epidemiology and risk between the 2 groups.

Our study has the expected limitations associated with a smaller cohort. Because we only included microbiologically confirmed infections, we have undoubtedly missed those infections such as pneumonia and skin and soft tissue infections for which organisms are infrequently identified. However, inclusion of only microbiologically defined infections likely reduced variability between reviewers in our retrospective study. We were also limited by inconsistent collection of lymphocyte subsets and immunoglobulin levels, precluding a more comprehensive assessment of immune reconstitution following CAR-T infusion. Additionally, the SARS-CoV-2 pandemic unfolded during the study, which may have reduced follow-up and available data for patients treated after March 2020. Importantly, we benchmarked our CD30 results against a concurrent cohort of CD19 CAR-T patients to ensure that our finding of higher viral infections was not only because of the timing of treatment during the SARS-CoV-2 pandemic. Finally, although the majority of patients returned to their local oncologist after the first 3 months following CAR-T therapy, all outside medical records available to the investigators were reviewed to limit any impact on data collection.

Given differences in infection risk following treatment, CD30 patients may benefit less than CD19 patients from antibacterial prophylaxis as they are less likely to develop severe neutropenia and bacterial infections. Antiviral prophylaxis against HSV and VZV likely remains important given multiple VZV infections following CD30 CAR-T in patients off prophylaxis. Although ours is a small cohort, no patients in our study developed fungal infections before relapse, suggesting that CD30 CAR-T patients may not require antifungal prophylaxis. Additional studies on the infectious risk of novel CAR T-cell therapies are needed to identify patients most likely to develop severe infections and to tailor antimicrobial prophylaxis to specific CAR T-cell therapies.

## Supplementary Material

ofaf541_Supplementary_Data

## Supplementary Data

Supplementary materials are available at *Open Forum Infectious Diseases* online. Consisting of data provided by the authors to benefit the reader, the posted materials are not copyedited and are the sole responsibility of the authors, so questions or comments should be addressed to the corresponding author.
