# Supplementary material for: Infectious Complications Following CD30 Chimeric Antigen Receptor T-cell Therapy in Adults
Source: Open Forum Infect Dis. 2025 Sep 18;12(9):ofaf541. doi: 10.1093/ofid/ofaf541 (PMC12449069; doi:10.1093/ofid/ofaf541)

# **SUPPLEMENTARY TABLES AND FIGURES**

**Figure S1**. **Greater number of viral infections were observed after CD30 CAR T-cell therapy compared to bacterial infections.** Cumulative incidence curves demonstrating time to first infections in the one year after CD30 CAR T-cell therapy, including all infections (A), only viral infections (B), and only bacterial infections (C).

**Alt text**: Cumulative incidence curve graphs of time to first infection after CD30 CAR T-cell therapy.

A.


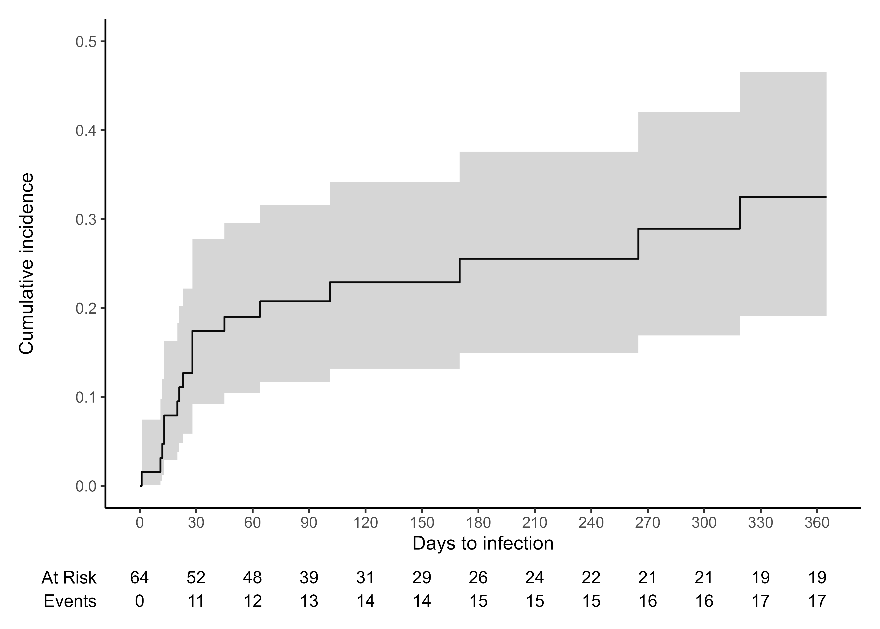


| **Characteristic** | **N** | **Day 28** | **Day 90** | **Day 180** | **Day 365** |
| --- | --- | --- | --- | --- | --- |
| Cumulative incidence % | 64 | 17% (9.2-28%) | 21% (12-32%) | 26% (15-38%) | 32% (19-47%) |

B.


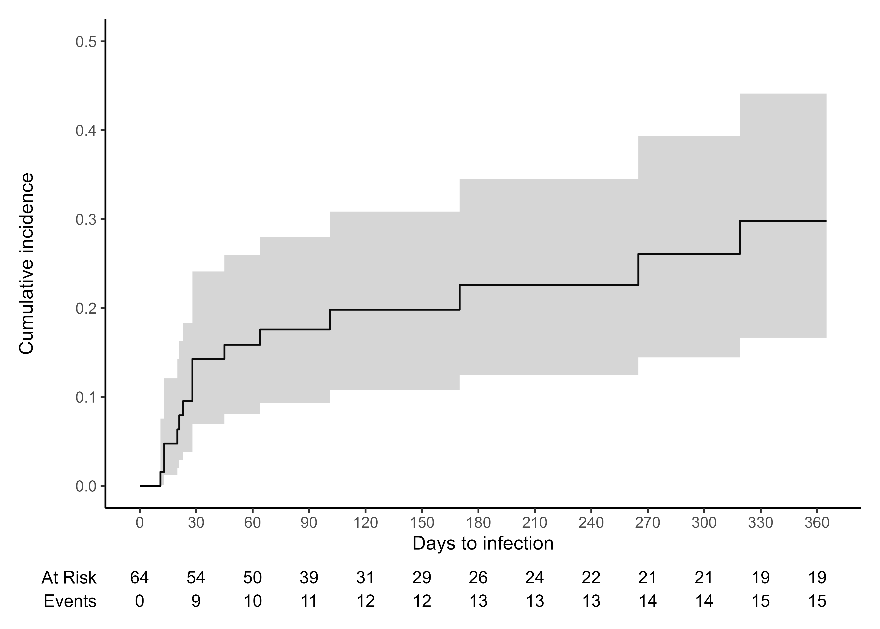


| **Characteristic** | **N** | **Day 28** | **Day 90** | **Day 180** | **Day 365** |
| --- | --- | --- | --- | --- | --- |
| Cumulative incidence % | 64 | 14% (7.0-24%) | 18% (9.3-28%) | 23% (12-35%) | 30% (17-44%) |

C.


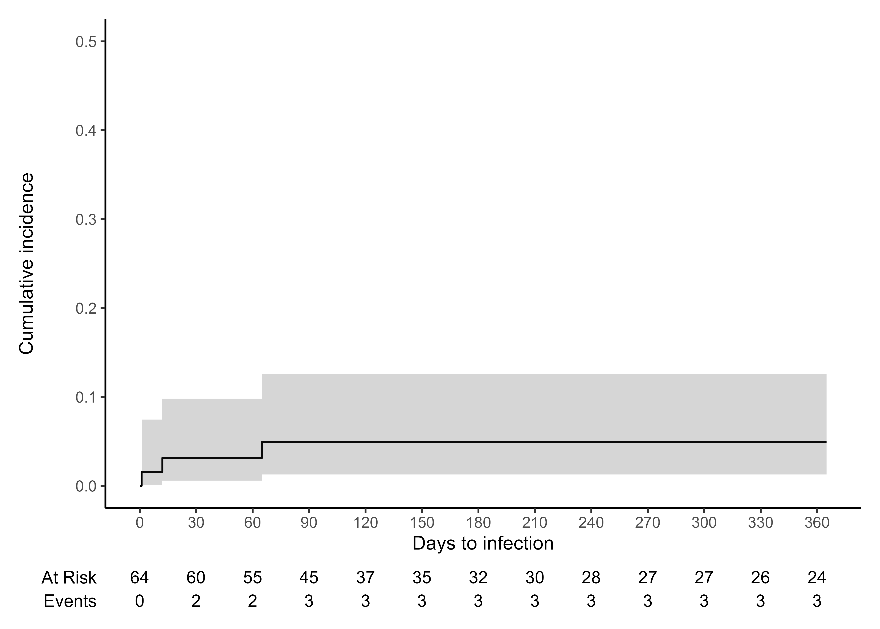


| **Characteristic** | **N** | **Day 28** | **Day 90** | **Day 180** | **Day 365** |
| --- | --- | --- | --- | --- | --- |
| Cumulative incidence % | 64 | 3.2% (0.58-9.8%) | 4.9% (1.3-13%) | 4.9% (1.3-13%) | 4.9% (1.3-13%) |

**Table S1.** Incidence and severity of infections after CD30 CAR T-cell infusion (19 infections, 17 patients)

| Type of Infection | Infection Days  0-28, n=64 | | Infection Days  29-90, n=52 | | Infection Days  91-365, n=39 | | Severity | | |
| --- | --- | --- | --- | --- | --- | --- | --- | --- | --- |
|  | Events | % | Events | % | Events | % | Grade 1 | Grade 2 | Grade 3 |
| Any Infection | **11** | **17%** | **3** | **6%** | **5** | **13%** | **15** | **3** | **1** |
| Bacterial Infections | **2** | **3%** | **1** | **2%** | **0** | **0** | **3** | **0** | **0** |
| Bloodstream | 0 | 0 | 1 | 2% | 0 | 0 | 0 | 1 | 0 |
| Skin | 2 | 3% | 0 | 0 | 0 | 0 | 2 | 0 | 0 |
| Viral Infections | **9** | **14%** | **2** | **4%** | **5** | **13%** | **13** | **2** | **1** |
| Respiratory Virus | 5 | 8% | 1 | 2% | 4 | 10% | 9 | 0 | 1 |
| Other (HSV, VZV, EBV) | 4 | 6% | 1 | 2% | 1 | 3% | 4 | 2 | 0 |

**Figure S2**. **Density and severity of infections after CD30 CAR T-cell therapy (HSCT/CAR T trial patients removed).** (A) Density and (B) severity of infections (15 patients with 16 infections), including bacterial and viral etiologies during the first 1 year following CD30 CAR T-cell therapy, broken into infection periods (0-28, 29-90, and 91-365 days). Data shown is censored for relapse. No fungal infections were observed.

**Alt text**: Graphs showing density and severity of bacterial and viral infections after CD30 CAR T-cells with HSCT/CART trial patients removed.

A.


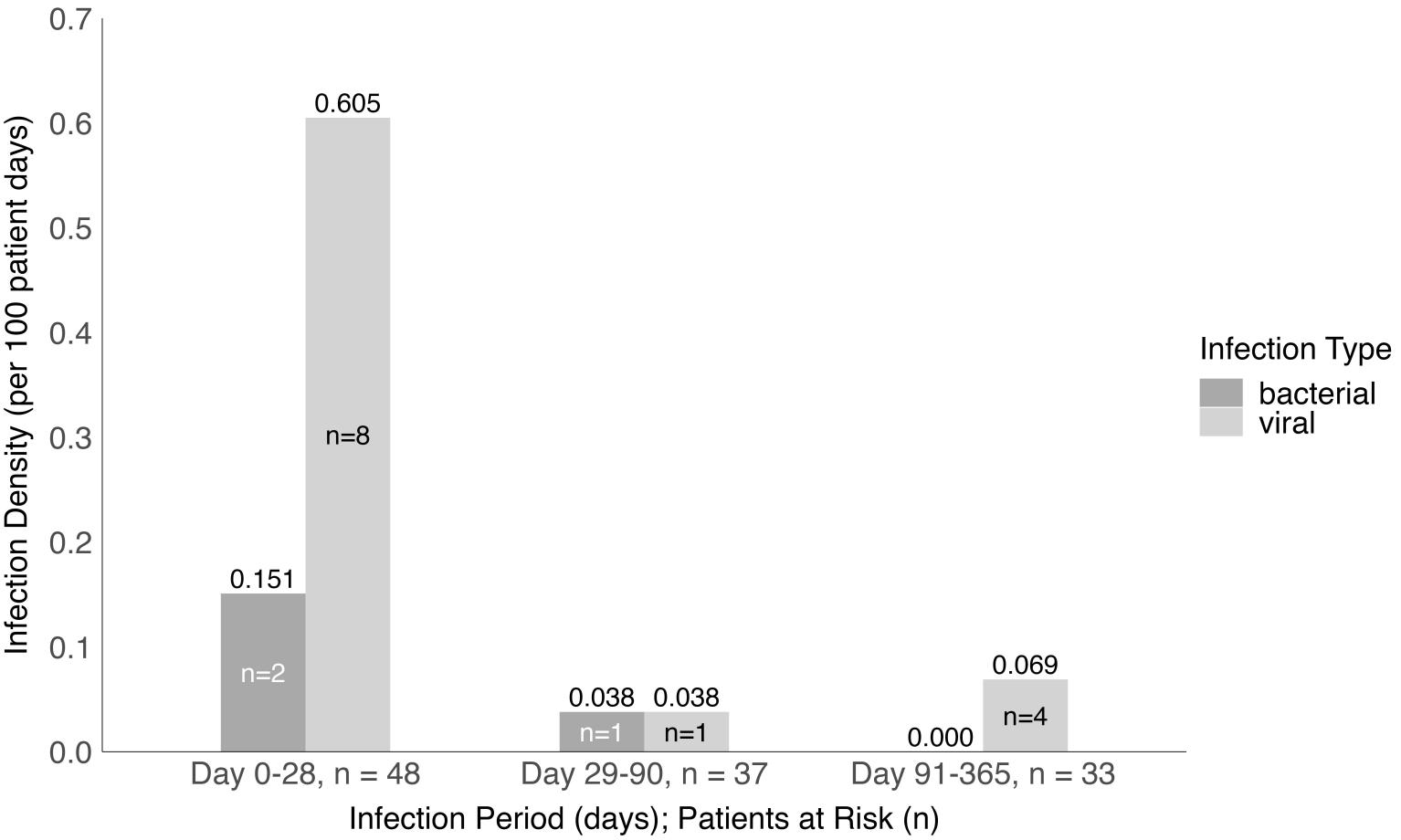


B.


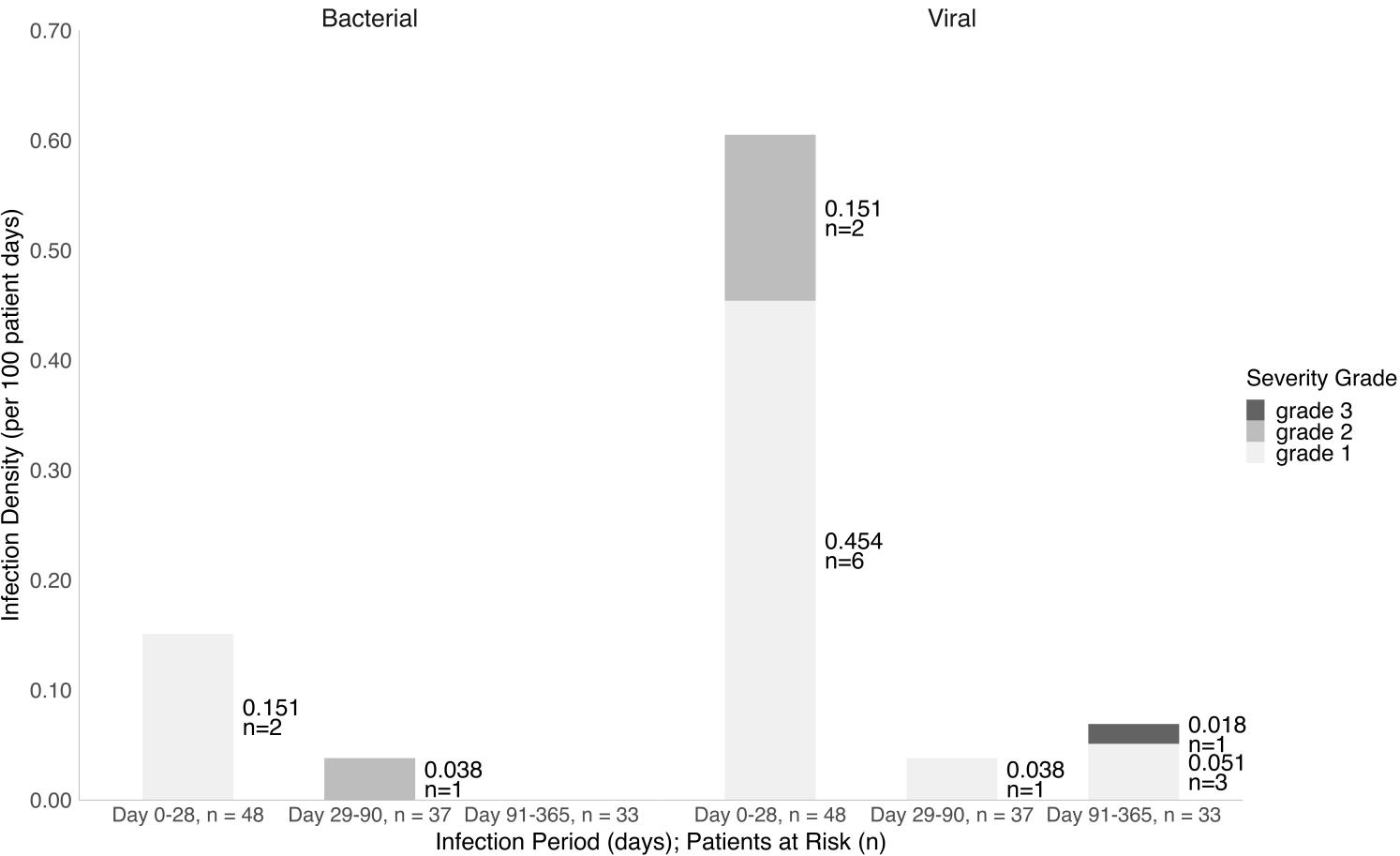


**Table S2**. Univariate association between patient variables and 1-year infection risk among CD30 CAR T-cell patients, estimated using a log binomial model. Continuous variables were compared above versus below the median.

| **Variable** | **Level** | **RR** | **Lower CI** | **Upper CI** |
| --- | --- | --- | --- | --- |
| Age (years) | <40 (Ref) | 1 |  |  |
|  | 40-59 | 0.33 | 0.08 | 1.13 |
|  | >=60 | 1.43 | 0.63 | 3.23 |
| Sex | Female (Ref) | 1 |  |  |
|  | Male | 0.59 | 0.26 | 1.31 |
| Race | White (Ref) | 1 |  |  |
|  | Black | 0.95 | 0.27 | 3.31 |
| Malignancy type | Peripheral T cell lymphoma (Ref) |  |  |  |
|  | Hodgkins lymphoma | 0.91 | 0.35 | 2.36 |
| Disease state pre-infusion | Complete remission (Ref) |  |  |  |
|  | Partial remission | 1.82 | 0.56 | 5.88 |
|  | Stable disease | 1.25 | 0.19 | 8.44 |
|  | Progressive disease | 1.38 | 0.48 | 3.97 |
| KPS score | <90 (median) | 0.97 | 0.41 | 2.28 |
| HCT-CI score | >2 (median) | 1.58 | 0.71 | 3.54 |
| Prior chemo lines | >4 (median) | 1.84 | 0.8 | 4.21 |
| HSCT before CAR T |  | 1.07 | 0.3 | 3.84 |
| HSCT type | Autologous (Ref) | 1 |  |  |
|  | Allogeneic | 0.6 | 0.09 | 3.76 |
| Days HSCT to CAR T | >1087 (median) | 1.5 | 0.62 | 3.65 |
| Corticosteroids (day-30 to 1 year post-infusion) |  | 1.29 | 0.56 | 2.99 |
| Antibiotic prophylaxis |  |  |  |  |
|  | Fluoroquinolone | 0.69 | 0.31 | 1.56 |
|  | Fluconazole | 0.82 | 0.36 | 1.85 |
|  | Bactrim | 0.66 | 0.28 | 1.56 |
|  | Valacyclovir | 0.8 | 0.33 | 1.92 |
| **ANC at Day -30** | **>3.4 (median)** | **0.31** | **0.11** | **0.84** |
| ALC (10^9^ cells/L) at Day -30 | >0.7 (median) | 1.45 | 0.64 | 3.27 |
| ANC at lymphodepletion | >3.2 (median) | 0.42 | 0.17 | 1.05 |
| ALC at lymphodepletion | >0.7 (median) | 2.21 | 0.93 | 5.25 |
| Days of lymphopenia <200 cells/mm | >7 (median) | 0.44 | 0.14 | 1.36 |
| Cytokine release syndrome |  | 1.64 | 0.72 | 3.71 |
| CRS grade max | Grade 0 (Ref) |  |  |  |
|  | Grade 1 | 1.59 | 0.62 | 4.06 |
|  | Grade 2 | 1.75 | 0.53 | 5.75 |

The following variables were not included in univariate analysis due to insufficient group size: Ethnicity, Lymphodepletion, Auto HSCT within 90 days of CAR T, Days of neutropenia, Pre-treatment infection, CRS treatment, Neurotoxicity, ICU admission, or Cause of death

Definitions: KPS, Karnofsky Performance Status; HCT-CI, Hematopoietic Stem Cell Transplant-specific Comorbidity Index; HSCT, Hematopoietic Stem Cell Transplantation; ANC, Absolute Neutrophil Count (x10^9^ cells/L); ALC, Absolute Lymphocyte Count (x10^9^ cells/L); CRS, Cytokine Release Syndrome

**Figure S3**. **Immune recovery after CD30 CAR T-cell therapy (HSCT/CAR T trial patients removed) demonstrates few differences between infected and uninfected patients within 1 year after infusion**. A) Absolute neutrophil counts (ANC) and B) absolute lymphocyte counts (ALC) at timepoints relative to the day of CAR-T infusion. Comparisons between infected and uninfected patients were performed using the Wilcoxon rank-sum test. Note: 3 ANC outlier values are missing from the plot (range 15.3-18) and 1 ALC outlier values are missing from the plot (14).

*P-value <0.05.

**Alt text**: Graphs showing neutrophil and lymphocyte counts over time after CD30 CAR T-cells with HSCT/CAR T trial patients removed


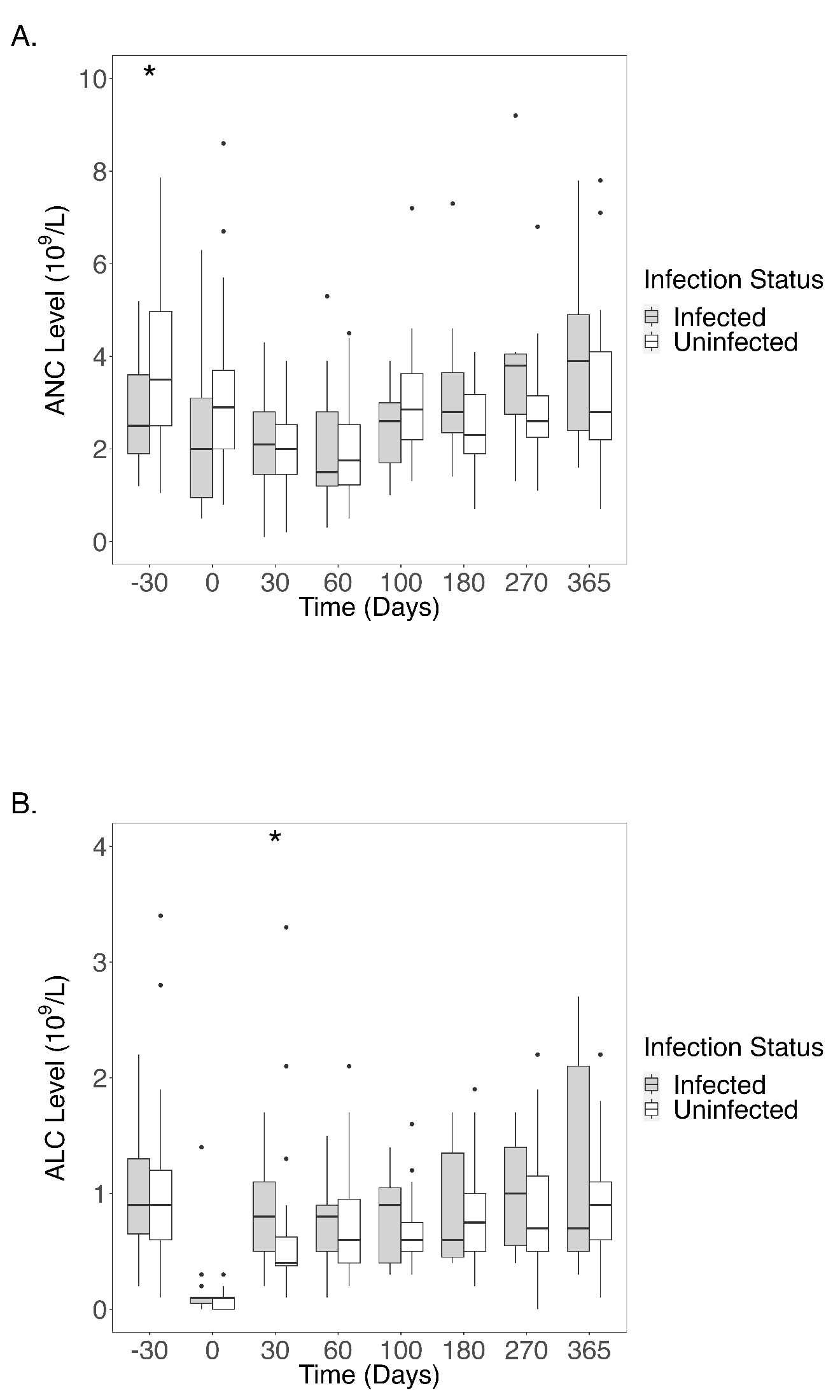


**Table S3.** Demographics of patients within one year after anti-CD19 CAR-T therapy.

| **Patient characteristics**  **N(%), Median[total range]** | **CD19**  **N = 50** |
| --- | --- |
| **Age** | 60.6 [21.5, 81.0] |
| **Sex (male)** | 31 (62.0) |
| **Race** |  |
| White | 37 (74.0) |
| Black | 9 (18.0) |
| Other | 4 (8.0) |
| **Ethnicity** |  |
| Hispanic | 5 (10.0) |
| Non-Hispanic | 45 (90.0) |
| Unknown | 0 (0) |
| **Malignancy** |  |
| Acute Lymphocytic Leukemia (ALL) | 12 (24.0) |
| Diffuse Large B-Cell Lymphoma | 30 (60.0) |
| Follicular or Mantle Cell Lymphoma | 8 (16.0) |
| **Disease state prior to CAR-T** |  |
| Complete Remission (CR) | 0 (0) |
| Partial Remission (PR) | 7 (14.0) |
| Very Good Partial Remission (VGPR) | 1 (2.0) |
| Stable Disease (SD) | 5 (10.0) |
| Progressive Disease (PD) | 36 (72.0) |
| Unknown | 1 (2.0) |
| **KPS score^1^** | 80.0 [60.0, 100.0] |
| **HCT-CI score^2^** | 4.0 [2.0, 14.0] |
| **Prior lines of chemotherapy** | 4.0 [3.0, 5.0] |
| **CAR-T lymphodepletion regimen** |  |
| Fludarabine/Bendamustine | 5 (10.0) |
| Fludarabine/Cyclophosphamide | 44 (88.0) |
| Bendamustine | 1 (2.0) |
| Post-HSCT infusion | 0 (0) |
| **Anti-CD19 product** | 50.0 (100.0) |
| Yescarta | 26 (52.0) |
| Tecartus | 1 (2.0) |
| Kymriah | 6 (12.0) |
| Anti-CD19 trial product | 17 (34.0) |
| **HSCT before CAR-T** | 14 (28.0) |
| Allogenic | 8 (16.0) |
| Autologous | 6 (12.0) |
| **Duration between HSCT and CAR-T (Days)** | 1404.5 [245.0, 9568.0] |
| **Auto HSCT within 90 days of CAR T-cell infusion** | 4 (8.0) |
| **HSCT within 1 year after CAR-T** | 5 (10.0) |
| **Corticosteroids^3^**  Before infection (n=9 patients with infection) | 22 (44.0)  6(66.7) |
| **Antimicrobial prophylaxis** |  |
| Fluoroquinolones | 45 (90.0) |
| Fluconazole | 44 (88.0) |
| Trimethoprim-sulfamethoxazole | 31 (62.0) |
| Valacyclovir | 45 (90.0) |
| **ANC at Day -30** | 3.2 [0.1, 19.3] |
| **ALC at Day -30** | 0.6 [0.0, 4.0] |
| **ANC at lymphodepletion^4^** | 2.8 [0.1, 14.1] |
| **ALC at lymphodepletion^5^** | 0.6 [0.0, 3.4] |
| **Total days of neutropenia^6^** | 14.0 [0.0, 228.0] |
| **Total days of lymphopenia^7^** | 9.5 [155.0, 3513.0] |
| **Pre-CAR-T infection by organism** |  |
| Bacterial | 5 (10.0) |
| Viral | 1 (2.0) |
| Fungal | 1 (2.0) |
| **Time in study (days)** | 116.5 [1.0, 365.0] |
| ^1^ KPS, Karnofsky Performance Status  ^2^ HCT-CI, Hematopoietic Stem Cell Transplant-specific Comorbidity Index  ^3^Steroids (30 days prior to CAR-T through 1 year after CAR-T) include dexamethasone, prednisone, and methylprednisolone  ^4^ ANC, absolute neutrophil count (x 10^9^ cells/L)  ^5^ ALC, absolute lymphocyte count (x 10^9^ cells/L)  ^6^ Neutropenia defined as ANC <0.5 x 10^9^ cells/L  ^7^ Lymphopenia defined as ALC <0.2 x 10^9^ cells/L |  |

**Table S4.** CAR T-related outcomes and toxicity in patients within one year after anti-CD19 CAR-T therapy

| **Patient characteristics**  **N(%), Median[total range]** | **CD19**  **N=50** |
| --- | --- |
| **Cytokine-release syndrome (CRS)** | 34 (68.0) |
| **CRS grade** |  |
| Grade 1 | 17 (34.0) |
| Grade 2 | 16 (32.0) |
| Grade 3 | 1 (2.0) |
| **CRS treatment** |  |
| Tocilizumab | 18 (36.0) |
| Tocilizumab + Steroids | 2 (4.0) |
| **Neurotoxicity (ICANS)** | 14 (28.0) |
| **ICANS grade** |  |
| Grade 1 | 3 (6.0) |
| Grade 2 | 3 (6.0) |
| Grade 3 | 6 (12.0) |
| Grade 4 | 2 (4.0) |
| **ICANS treatment** |  |
| Steroids | 11 (22.0) |
| Steroids + Rimiducid (kill switch) | 1 (2.0) |
| **ICU Admission (within 30 days of CAR-T infusion)** | 13 (26.0) |
| **Relapse within 1 year after CAR T-cell therapy** | 28 (56.0) |
| **30-day all-cause mortality** | 0 (0) |
| **1-year all-cause mortality*** | 20 (40.0) |
| **Cause of 1-year mortality**** |  |
| Infection-related | 5 (25.0) |
| Relapse-related | 15 (75.0) |
| Other | 2 (10.0) |

*Mortality regardless of relapse status

**Two-patients had two causes of death listed (both relapse and infection)

**Table S5. Microbiologically-confirmed infections within 1-year after CD19 CAR T-cell infusion.** Type and number of bacterial and viral infections within 1 year after CD19 CTI are shown, censored for relapse. Number in parathesis indicates total number of infections for causative organism. No fungal infections were observed.

| **Type of infection** | **Organism** |
| --- | --- |
| Bloodstream infection | *Enterococcus faecalis** |
|  | *Staphylococcus epidermidis* |
|  | *Streptococcus mitis* |
| Urinary tract infection | *Escherichia coli* |
| Colitis | *Clostridioides difficile* (2) |
| Respiratory tract infection |  |
| Upper tract | Rhinovirus |
|  | RSV |
| Lower tract | SARS-CoV-2 |

* *Enterococcus faecalis* endocarditis

Note: HSV=Herpes Simplex Virus; RSV=Respiratory Syncytial Virus

**Figure S4.** **More frequent bacterial infections of greater severity were observed in the first 28 days after CD19 CAR T-cell therapy.** Density (A) and severity (B) of infections occurring during the first year following CAR T-cell therapy, broken into infection periods (0-28, 29-90, and 91-365 days). No fungal infections were observed in either group. Data shown is censored for relapse.

**Alt text**: Graphs showing density and severity of bacterial and viral infections after CD19 CAR T-cells.

A.


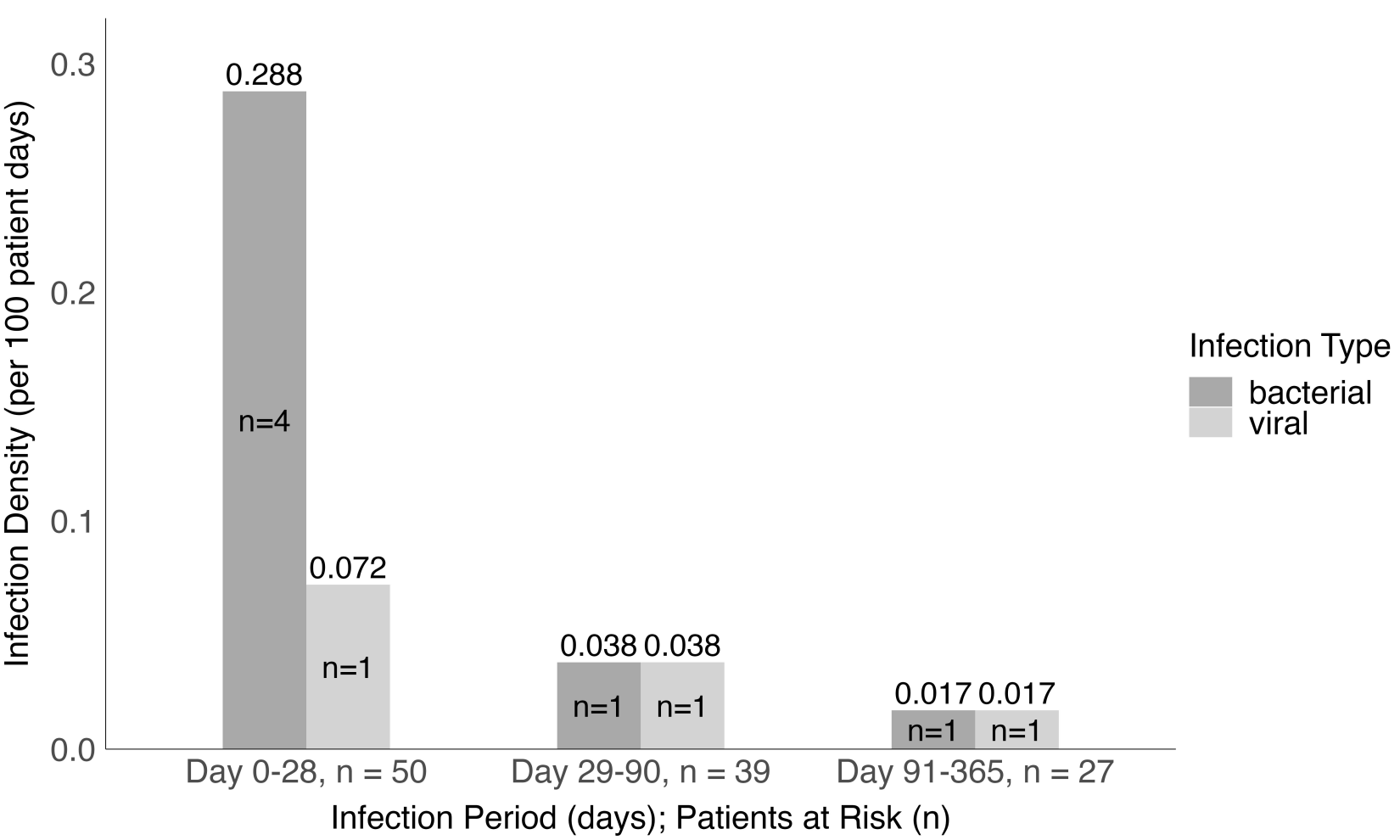


B.


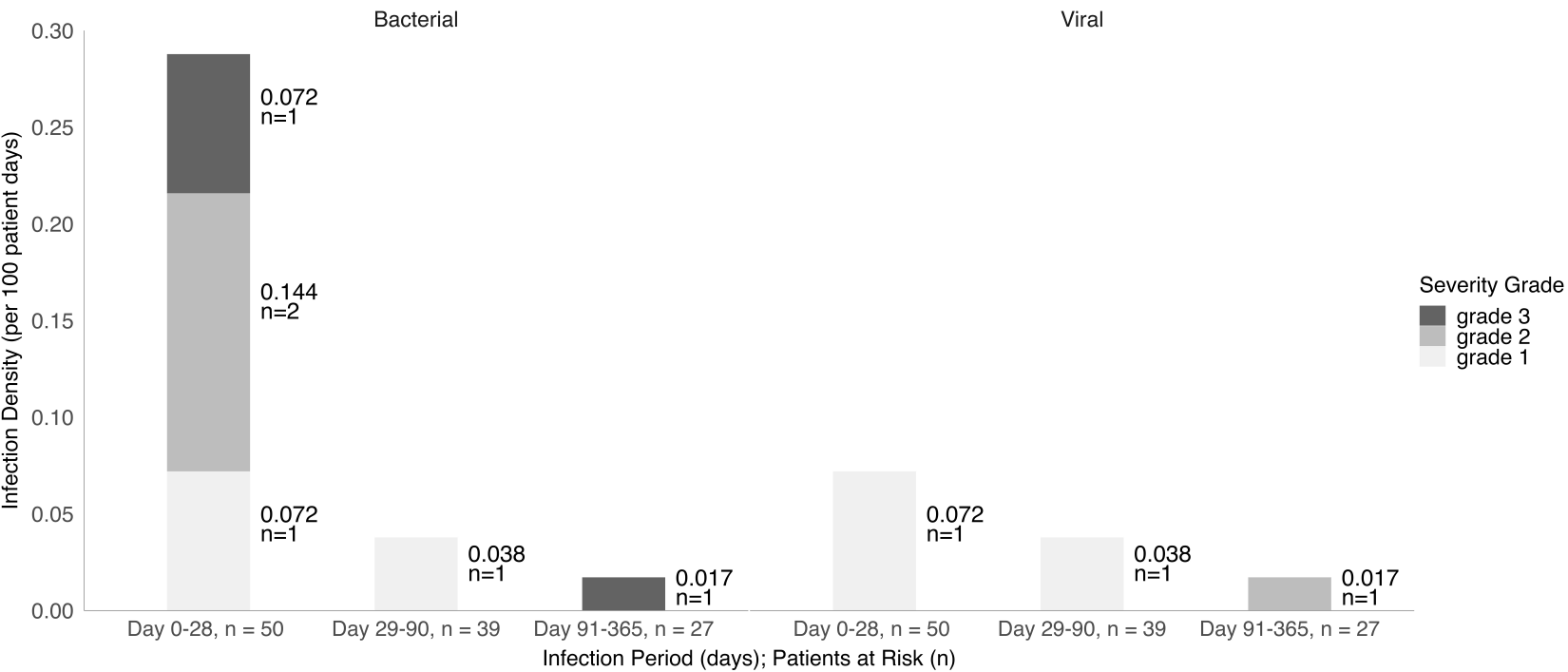


**Figure S5**. **Immune recovery after CD19 CAR T-cell therapy demonstrates few differences between infected and uninfected patients within 1 year after infusion**. A) Absolute neutrophil counts (ANC) and B) absolute lymphocyte counts (ALC) at timepoints relative to the day of CAR-T infusion. Comparisons between infected and uninfected patients were performed using the Wilcoxon rank-sum test. Note 3 ANC outlier values are missing from the plot (range 10.2-19.3).

*P-value <0.05, **p-value <0.01.

**Alt text**: Graphs showing neutrophil and lymphocyte counts over time after CD19 CAR T-cells.


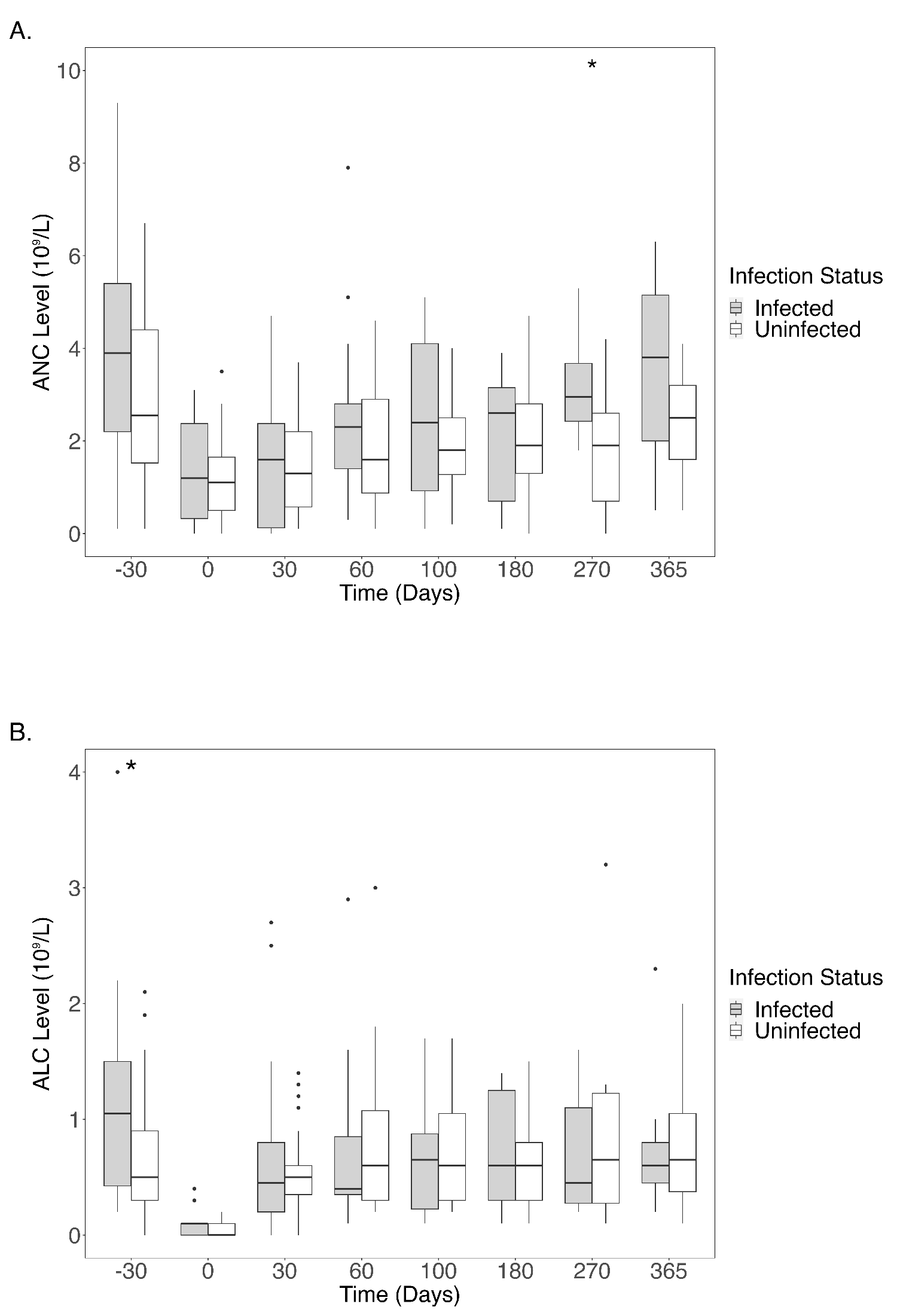

Supplement: ofaf541_Supplementary_Data [file ofaf541_supplementary_data.zip › CD30 Supplemental Figures 2025_07_25_TA.docx]
